# Symptom evolution, persistence, and pharmacological management in paediatric NMDA receptor antibody encephalitis

**DOI:** 10.1016/j.bbih.2026.101320

**Published:** 2026-07-31

**Authors:** Alessandro Santagostino Barbone, Yoshua Collins-Sawaragi, Maria Margherita Mancardi, Roberta Biddeci, Chiara Scaroni, Claire Thompson, Silvia Buratti, Lino Nobili, Ming Lim, Elisa De Grandis, Thomas Rossor, Michael Eyre

**Affiliations:** a Department of Neuroscience, Rehabilitation, Ophthalmology, Genetic and Maternal Infantile Sciences, University of Genoa, Genova, Italy; b Children's Neurosciences, Evelina London Children's Hospital at Guy's and St Thomas' NHS Foundation Trust, London, UK; c Department of Paediatric Neurology, Birmingham Children's Hospital, Birmingham, UK; d Department of Clinical Paediatrics Sciences, Unit of Child Neuropsychiatry, Member of the ERN Epicare Network, IRCCS Istituto Giannina Gaslini, Genova, Italy; e Neonatal and Paediatric Intensive Care Unit, Emergency Department, IRCCS Giannina Gaslini, Genova, Italy; f Department of Women and Children's Health, School of Life Course Sciences (SoLCS), King's College London, London, UK; g Department of Neurology, Hunan Children's Hospital, Changsha, China; h Department of Imaging Physics & Engineering and Department of Early Life Imaging, School of Biomedical Engineering & Imaging Sciences, King's College London, London, UK

**Keywords:** NMDAR antibody, Encephalitis, Paediatric, Symptoms, Symptomatic treatment

## Abstract

**Objective:**

To characterise symptom onset, progression, and persistence in paediatric N-methyl-D-aspartate receptor antibody encephalitis (NMDARE) using the Paediatric Autoimmune encephalitis Severity Scale (PASS), and to describe real-world symptomatic pharmacological management.

**Methods:**

We performed a retrospective study of children (<18 years) with confirmed NMDARE admitted to two tertiary centres (2012-2024). Disease severity was assessed using the modified Rankin Scale (mRS) and PASS at nadir, discharge, one year, and last follow-up. Symptomatic treatments and their timings, clinician-rated benefits and adverse events were recorded, with adverse event:benefit ratios calculated.

**Results:**

Thirty-five patients were included (median age 8 years [IQR 4–13]; 71% female). Median peak severity was mRS 5 and PASS 22/30. Seizures and psychiatric manifestations were the most frequent presenting symptoms, whereas movement disorders, motor deficits, and autonomic dysfunction typically appeared later. Younger children (<12 years) presented with a more florid neurological picture, whereas older patients more often presented with psychiatric features; both groups progressed to similar multisystem severity at nadir. At one year, 68% achieved mRS 0–1 (median PASS 3/30), increasing to 87% at last follow-up (median 5 years). PASS identified persistent impairments in activities of daily living (26%), speech/communication (17%), and sleep (14%) at last follow-up. Symptom-directed treatments showed high perceived benefit across drug classes; second-generation antipsychotics were generally well tolerated, except risperidone, which showed higher adverse event rates.

**Interpretation:**

Persistent neurocognitive and functional deficits are common following NMDARE, captured better by PASS than mRS. Symptom-directed treatments showed overall favourable tolerability, with second-generation antipsychotics except risperidone demonstrating good safety.

## Highlights

• Early symptom patterns vary by age, but severity converges at disease peak.
• Persistent cognitive and daily function deficits common after encephalitis.
• Speech and sleep disturbances are the most frequent long-term residual symptoms.
• PASS score detects deficits missed by global disability scales.
• Symptom-directed drugs effective; most antipsychotics well tolerated.

## 1 Introduction

N-methyl-D-aspartate receptor antibody encephalitis (NMDARE) is the most common autoimmune encephalitis in children (Chen et al., 2024). In experimental models, autoantibody binding to the NMDAR causes receptor internalization and impairment of long-term synaptic plasticity resulting in memory deficits, seizures, anhedonia, and depressive behaviours (Planagumà et al., 2015; Li et al., 2015; Wright et al., 2015). In patients, NMDARE presents as a severe polysymptomatic encephalopathic syndrome with prominent psychiatric features, speech disturbances, cognitive impairment, seizures, movement disorders, dysautonomia, and sleep abnormalities. The presentation varies by age: prepubertal children tend to exhibit more florid movement disorders, while postpubertal patients present more often with pronounced neuropsychiatric symptoms (Armangue et al., 2013).

Clinical outcomes have mostly been assessed with the modified Rankin Scale (mRS), however recent studies have emphasized the limitations of this scale in capturing long-term neurocognitive sequelae in both adult and paediatric populations due to its predominant focus on motor disability (De Bruijn et al., 2018; Flet-Berliac et al., 2023; Brenner et al., 2024). In view of the polysymptomatic nature of NMDARE, alternative outcome measures have been developed and validated, including the Clinical Assessment Scale in Autoimmune Encephalitis (CASE) for adults (Lim et al., 2019) and, more recently, the Paediatric Autoimmune encephalitis Severity Scale (PASS) for children (Madaan et al., 2026).

Management of paediatric NMDARE in the acute phase is particularly challenging and typically requires a coordinated multidisciplinary approach. Even with prompt initiation of immunotherapy, symptom progression is common during the first weeks of the illness. Approximately 70% of patients require admission to the intensive care unit (ICU) for airway protection, severe dyskinesias, autonomic instability, fluctuating levels of consciousness, or respiratory dysfunction (Titulaer et al., 2013; De Montmollin et al., 2017). First-line immunotherapy generally consists of high-dose corticosteroids, intravenous immunoglobulin (IVIG), and/or plasma exchange. Second-line immunotherapy, most commonly rituximab, is frequently used when response to first-line immunotherapy is inadequate or transient (Nosadini et al., 2021a). Despite advances in immunotherapy, evidence guiding symptom-directed management during the acute phase is limited, and strategies to address symptomatic control are not well established (Mohammad et al., 2016). In this study we aimed to map the evolution and persistence of symptoms over time using the novel PASS scale and describe the use and effects of symptom-directed pharmacological interventions in paediatric NMDARE.

## 2 Methods

### 2.1 Study design and participants

This retrospective observational cohort study included children (age < 18 years) with confirmed anti-NMDAR encephalitis diagnosed based on clinical presentation and positive anti-NMDAR serum antibody and/or cerebrospinal fluid (CSF), in accordance with established diagnostic criteria (Cellucci et al., 2020). Patients eligible for inclusion presented between 2012 and 2024 to two tertiary paediatric centres: Evelina London Children's Hospital (London, UK) and Gaslini Institute (Genoa, Italy). All patients underwent electroencephalography (EEG), magnetic resonance imaging (MRI), lumbar puncture with CSF analysis, and comprehensive bacterial and viral studies including herpes simplex virus (HSV). Anti-NMDAR antibody positivity was confirmed in respective regional centres with cell-based assays. Tumour screening included pelvis and lower abdomen ultrasonography, pelvis MRI or computed tomography.

### 2.2 Standard protocol approvals, registrations, and patient consents

As only data required for standard medical care were collected, a full ethics review under the terms of the Governance Arrangements of Research Ethics Committees was deemed not necessary by the study team.

### 2.3 Clinical data

Detailed clinical characteristics, ancillary diagnostic tests, treatments, and outcomes of enrolled patients were retrospectively reviewed by paediatric neurologists at each site using standardized data collection forms and entered in a secure database. Disease severity and clinical outcomes were assessed using the mRS and PASS at nadir, discharge, one-year follow-up and last recorded follow-up.

The PASS comprises 10 items, each scored 0–3 (total range 0–30): (1) psychiatric manifestations (delusions, hallucinations, obsessions, compulsions, mood lability, anxiety, autistic features); (2) excitement, agitation or restlessness; (3) movement disorder (dystonia, chorea, ballismus, stereotypies, ataxia); (4) seizures and epilepsy; (5) mental state (awareness, orientation, memory); (6) speech and communication dysfunction; (7) sleep dysfunction; (8) autonomic dysfunction (cardiovascular, gastrointestinal, respiratory, urinary, sweating, temperature regulation); (9) feeding; (10) safety and age-appropriate activities of daily living. For all items, a score of 0 indicates no symptoms/normal function, 1 indicates mild or intermittent symptoms/dysfunction, 2 indicates moderately disabling or frequent symptoms requiring intervention, and 3 indicates severe, continuous, or refractory symptoms requiring intensive supervision, full assistance, or posing risk of harm to self or others. Intubated/comatose patients score 3 by default for the psychiatric, agitation, movement disorder, speech/communication and sleep items.

### 2.4 Symptomatic treatments

Symptomatic medications were grouped into five main categories based on clinical indication or mechanism of action: anti-seizure medications (ASMs), antipsychotics (APs), benzodiazepines (BDZs), movement disorder directed drugs (MDDs) and anaesthetic agents (ANs).

The impression of benefit for each medication was evaluated retrospectively by a clinician not directly involved in patient clinical care and decision-making, based on documentation in the medical records, and classified as “Yes Benefit,” “No Benefit,” or “Unclear Benefit”. Adverse events were graded according to the National Institutes of Health Common Terminology Criteria for Adverse Events (CTCAE) version 5.0. A descriptive adverse effect–benefit ratio was calculated for each medication, defined as the number of patients experiencing adverse events divided by the number experiencing clinical benefit.

### 2.5 Statistical analysis

Descriptive statistics were used to compare age groups: younger (<12 years) and older (12–18 years). Between-group comparisons were performed using the Mann–Whitney *U* test for continuous variables and the chi-square or Fisher's exact test for categorical variables, as appropriate. Post hoc analyses examined associations between time to first immunotherapy and clinical outcome using Mann–Whitney U tests (days to first immunotherapy compared between good [mRS 0–1] and poor [mRS 2–6] outcome groups at last follow-up; and PASS score and length of hospital stay compared between patients receiving early [≤14 days] versus late [>14 days] immunotherapy) and Spearman rank correlation (days to first immunotherapy versus PASS score and length of hospital stay). Statistical analyses were performed using Python, version 3.10, and statistical significance was defined as p < 0.05.

## 3 Results

### 3.1 Patient characteristics

The cohort included 35 NMDARE patients, 25 female (71%) (Table 1). The median age at disease onset was 8 years (range 1 - 17); 23 (66%) were younger than 12 years. Twelve patients (34%) presented a prodromal event, including upper respiratory tract infection, diarrhoea and vomiting. Three patients (9%), all younger than 12 years, had a prior HSV-1 encephalitis. Four patients (11%, 2 < 12 years and 2 > 12 years) had an associated tumour (ovarian teratoma), which was resected in all cases.

**Table 1:** Patient characteristics.

Feature	All Patients (n = 35)	Patients <12 years (n = 23)	Patients ≥12 years (n = 12)	p-value
Sex				
Female	25 (71%)	17 (74%)	8 (67%)	0.71
Male	10 (29%)	6 (26%)	4 (33%)	
**Patient Age (years)**				
Mean (SD)	8 (5)	5 (3)	14 (1)	N/A
Median (IQR)	8 (4-13)	5 (2-8)	14 (14-15)	
Range	1 - 17	1 - 11	12 - 17	
**Ethnicity**				
Black	7 (20%)	5 (22%)	2 (17%)	1
Asian	7 (20%)	5 (14%)	2 (6%)	
Hispanic or Latin American	2 (6%)	1 (4%)	1 (8%)	
Mixed/Other	3 (9%)	2 (9%)	1 (8%)	
White Caucasian	16 (46%)	10 (43%)	6 (50%)	
**Prodromal event**				
Prodromal infectious event	12 (34%)	10 (43%)	2 (17%)	0.15
Prior HSV-1 encephalitis	3 (9%)	3 (13%)	0 (0%)	0.54
Tumour associated	4 (11%)	2 (9%)	2 (17%)	0.59
**Brain MRI**				
Abnormal	19 (54%)	13 (56%)	6 (50%)	0.74
**EEG**				
Abnormal	34 (97%)	23 (100%)	11 (92%)	0.34
Epileptic activity	11 (31%)	9 (39%)	2 (17%)	0.26
Extreme delta brush	2 (6%)	0 (0%)	2 (17%)	0.11
**CSF**				
Pleocytosis	17 (57%)	11 (55%)	6 (60%)	1
Elevated protein	6 (20%)	4 (20%)	2 (20%)	1
CSF-restricted oligoclonal bands	13 (43%)	8 (40%)	5 (50%)	0.73
**ICU**				
ICU admission	19 (54%)	12 (52%)	7 (58%)	1
**Number of days in ICU**				
Mean (SD)	33 (48)	28 (40)	42 (61)	0.31
Median (IQR)	11(4-40)	7 (4-34)	17(11-40)	
Range	1 - 176	1 - 119	1 - 176	
Mechanical ventilation	15 (43%)	8 (35%)	7 (58%)	0.28
**Super Refractory Status Epilepticus (SRSE)**				
SRSE occurred	6 (17%)	4 (17%)	2 (17%)	0.74
SRSE duration (days)				
Mean (SD)	22 (38)	32 (50)	6 (6)	0.8
Median (IQR)	4 (3-10)	4 (3-47)	6 (4-8)	
Range	2 - 90	3 - 90	2 - 10	
**Length of hospital stay (days)**				
Mean (SD)	75 (76)	69 (52)	88 (111)	1
Median (IQR)	60(30-89)	62(27-89)	55(41-76)	
Range	8 - 430	8 - 210	22 - 430	
**Last Follow-Up (Years)**				
Mean (SD)	5 (3)	6 (4)	3 (2)	0.07
Median (IQR)	4 (2-8)	6 (2-9)	3 (2-4)	
Range	0.6 - 13	0.8 - 13	0.6 - 8	
**Relapsing course**	6 (17%)	6 (26%)	0 (0%)	0.07

Abbreviations: SD standard deviation; IQR interquartile range; HSV-1 herpes simplex virus 1; SRSE super refractory status epilepticus; CSF cerebrospinal fluid; MRI magnetic resonance imaging. Data are n (n %) or mean (SD) median (IQR), range.

Brain MRI abnormalities were observed in 19/35 patients (54%). EEG abnormalities were present in 34 patients (97%), including epileptic abnormalities in 11 (31%), and extreme delta brush in 2 patients (6%), both older than 12 years. CSF revealed pleocytosis (>5 cells/μL) in 17/30 patients (57%), elevated protein levels in 6/30 patients (20%) and the presence of CSF-restricted oligoclonal bands in 13/30 patients (43%). There were no significant differences in characteristics between age groups.

### 3.2 Symptom onset and progression

The temporal sequence of symptom onset is shown in Fig. 1. Early-onset symptoms (median presentation within the first week) included predominantly cognitive and psychiatric features, as well as seizures and fever. In contrast, neurological symptoms such as movement disorders, motor deficits, and autonomic instability typically appeared later, with a median onset after the first week. Notably, seizures and psychiatric manifestations had a median time of presentation corresponding to disease onset, indicating that these two symptoms were the most frequent at initial presentation.

**Fig. 1:**
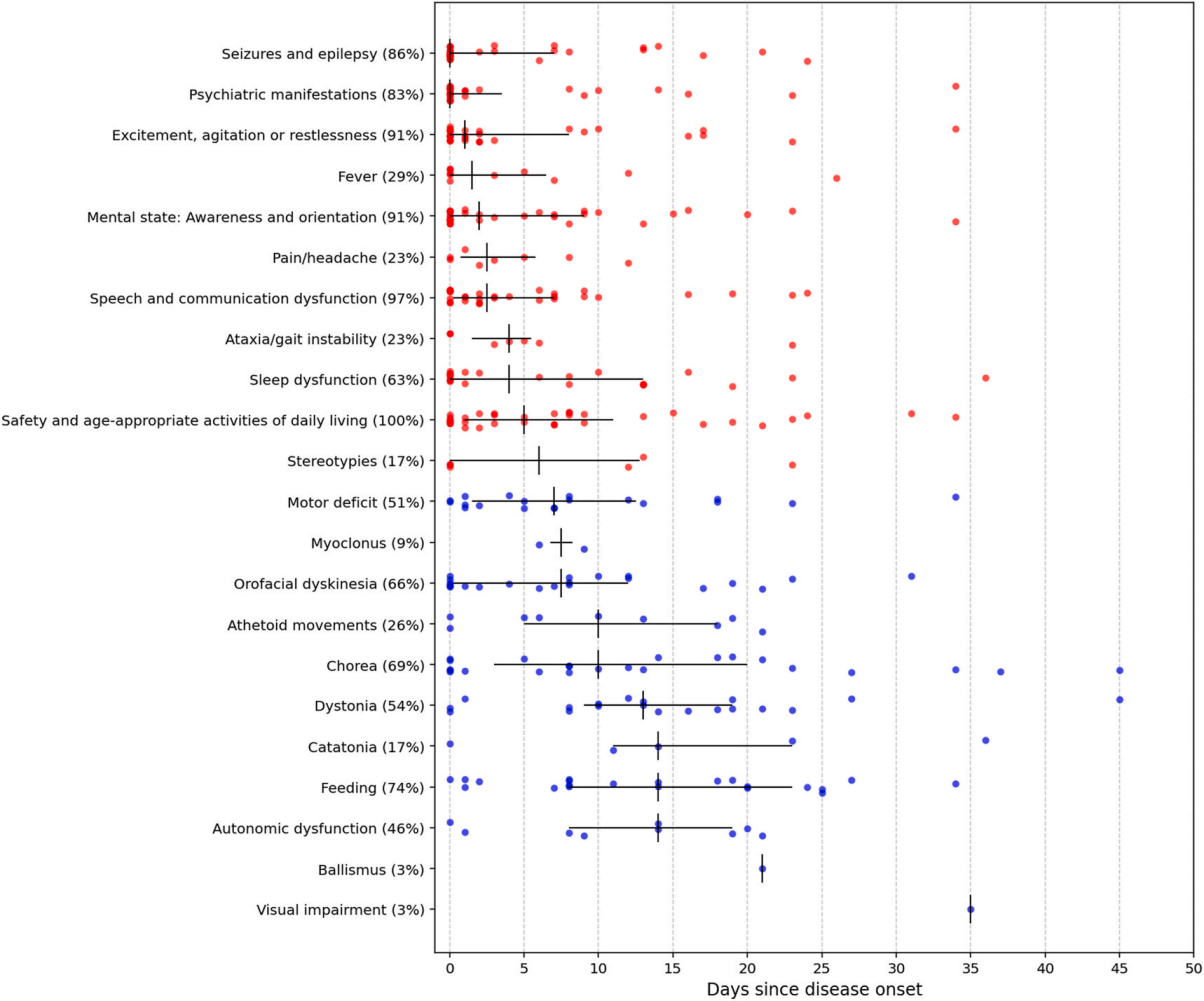
Time of first appearance for different symptoms. Numbers in brackets indicate the total percentage of patients presenting the symptom. Every dot represents a patient. In red are shown the symptoms with median (interquartile IQR) onset occurring within the first week of disease, in blue the symptoms with median (IQR) onset after the first week. (For interpretation of the references to color in this figure legend, the reader is referred to the Web version of this article.)

**Fig. 2:**
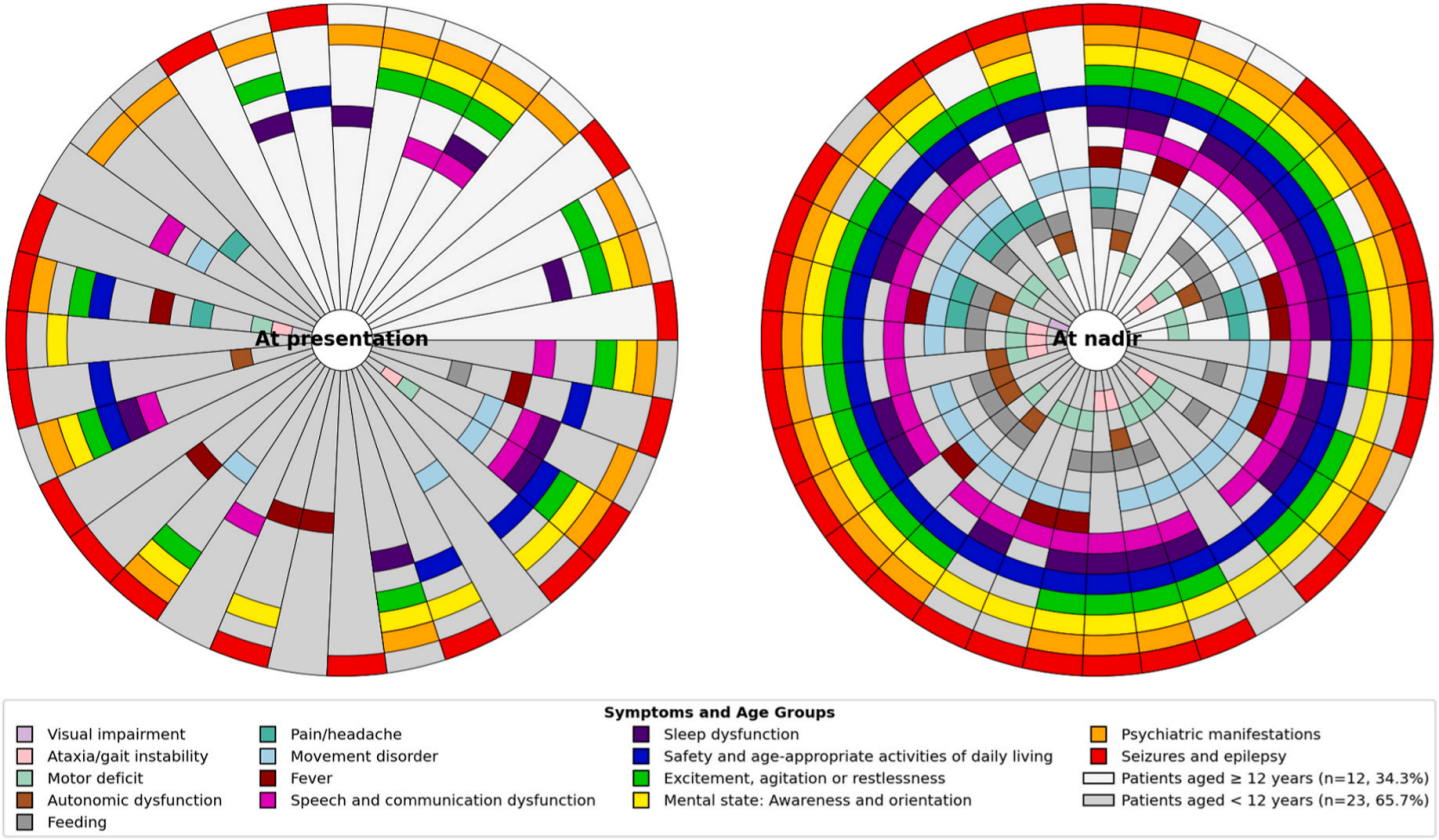
Patient symptom profile at presentation and at nadir (maximum disease severity). Each sector (wedge) represents one patient and each concentric ring one symptom domain, colour-coded as in the legend. Patients ≥12 years old at onset are shown in the section with white background, and those <12 in the section with grey background. Each patient occupies the same angular position in both panels. (For interpretation of the references to color in this figure legend, the reader is referred to the Web version of this article.)

Symptom presentation and progression according to age is shown in Fig. 2 and Table 2. Patients younger than 12 years tended to present more floridly, with a median of three symptoms at disease onset involving a broader spectrum of neurological, cognitive, and psychiatric domains, versus two in older patients (p = 0.06). This often resulted in limitations in age-appropriate daily activities from day of onset. In contrast, older patients typically presented with prominent psychiatric features and agitation, with fewer seizures and less interference with daily functioning initially. No patients older than 12 years presented with fever or neurological symptoms such as movement disorders, motor deficits, or gait instability at disease onset.

**Table 2:** Symptom presentation and evolution.

Symptom	Patients <12 years (n = 23)		Patients ≥12 years (n = 12)	
	Presentation n(%)	Nadir n(%)	Presentation n(%)	Nadir n(%)
**Psychiatric manifestations**	9 (39%)	17 (74%)	7 (58%)	10 (83%)
**Seizures and epilepsy**	12 (52%)	20 (87%)	4 (33%)	10 (83%)
**Mental state: Awareness and orientation**	9 (39%)	21 (91%)	4 (33%)	9 (75%)
**Excitement, agitation or restlessness**	6 (26%)	18 (78%)	6 (50%)	11 (92%)
**Speech and communication dysfunction**	6 (26%)	21 (91%)	2 (17%)	9 (75%)
**Sleep dysfunction**	4 (17%)	13 (56%)	4 (33%)	9 (75%)
**Safety and age-appropriate activities of daily living**	7 (30%)	23 (100%)	1 (8%)	12 (100%)
**Movement disorder**	5 (22%)	21 (91%)	0 (0%)	9 (75%)
**Fever**	5 (22%)	6 (26%)	0 (0%)	4 (33%)
**Motor deficit**	2 (9%)	13 (56%)	0 (0%)	5 (42%)
**Pain/headache**	2 (9%)	4 (17%)	0 (0%)	4 (33%)
**Ataxia/gait instability**	2 (9%)	6 (26%)	0 (0%)	1 (8%)
**Autonomic dysfunction**	1 (4%)	6 (26%)	0 (0%)	3 (25%)
**Feeding**	1 (4%)	14 (61%)	0 (0%)	8 (67%)
**Visual impairment**	0 (0%)	1 (4%)	0 (0%)	0 (0%)
**Mean (Median) [Range] Number of symptoms**	3 (3) [1-10]	9 (9) [4-12]	2 (2) [1-5]	9 (9) [3-12]

**Fig. 3:**
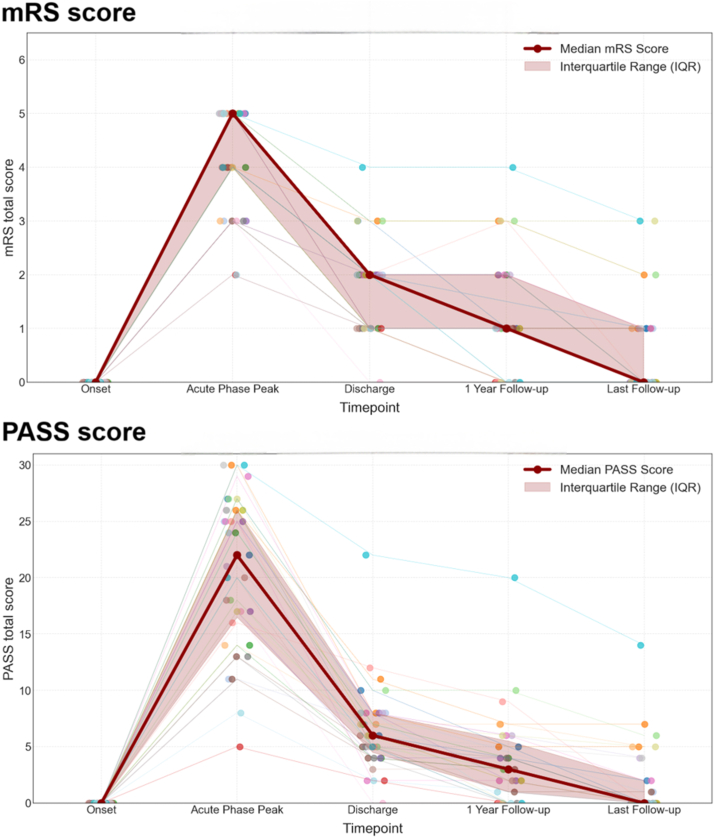
Longitudinal disease severity scores. Abbreviations: mRS, modified Rankin Scale; PASS, Pediatric Autoimmune encephalitis Severity Scale. Thin lines with corresponding dots at timepoints show individual patient score trajectories over time. The median and interquartile range for the whole cohort is shown by the thick line and shaded region.

**Fig. 4:**
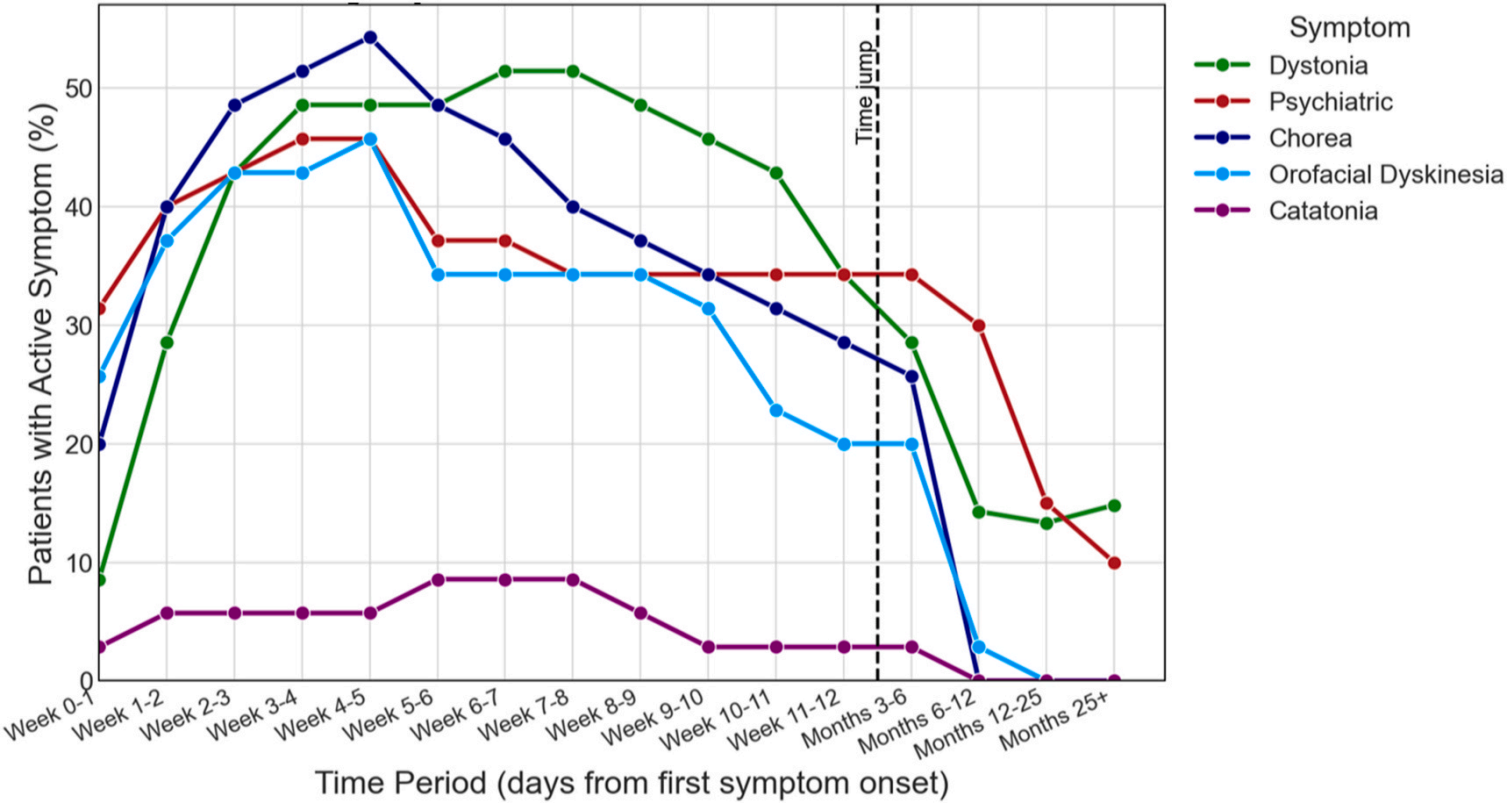
Psychiatric symptoms and movement disorder evolution. Each colored line with dots represents a different symptom. Color legend is shown in panel. Dashed black line marks a break in the x-axis scale, from weeks to months from disease onset. (For interpretation of the references to color in this figure legend, the reader is referred to the Web version of this article.)

The median interval from symptom onset to hospital admission in patients younger than 12 years was 7 days (range 0–34), trending towards slightly later in the older age group (median 10 days [range 0-116]) (p = 0.11). The median time from onset to nadir (maximum disease severity) was 16 days (IQR 9–25). Most patients converged towards a similar symptom spectrum at nadir regardless of age, with a median 9 symptoms for both age groups (p = 0.63) (Fig. 2, Table 2) and significant disease severity (but no deaths), with 51% of patients having mRS 5 (IQR 4-5, range 2-5). PASS scoring provided finer severity discrimination, with a median peak score of 22/30 (IQR 15–26, range 5–30) (Fig. 3). Nineteen patients (54%) were admitted to ICU with a median ICU stay of 11 days (IQR 4-40). Mechanical ventilation was required in 15 patients (43%). Six patients (17%) met criteria for super-refractory status epilepticus, as defined by the International League Against Epilepsy (Hirsch et al., 2018). Fig. 4 shows the trajectories of other core symptoms: chorea and orofacial dyskinesia peaked at weeks 4-5 and resolved within 6-12 months, while dystonia peaked later (weeks 7-8) and persisted longer in a minority of patients. Psychiatric symptoms peaked earlier (weeks 3-4) but also persisted longest.

### 3.3 Immunotherapy

First-line immunotherapy was administered in 33 patients (94%). The median time to first immune treatment was 16 days from disease onset (IQR 10-25, range 6-83). Treatments included, alone or in combination, intravenous immunoglobulins (IVIG) in 29 (83%), intravenous (IV) steroid pulse in 27 patients (77%), plasma exchange (PLEX) in 17 (49%) and high-dose oral steroid pulse in 4 (11%). Post hoc analyses found no significant associations between time to first immunotherapy and functional outcome (mRS), PASS score at last follow-up, or length of hospital stay (Supplementary Table 4).

Twenty-seven patients (77%) received oral steroid tapering, and 11 patients (31%) received a maintenance immunotherapy other than steroid tapering, including mycophenolate mofetil (n = 3), everolimus (n = 3), monthly IVIG (n = 3), and monthly pulse steroids (n = 2).

One or more second-line immunotherapies were administered to 20 patients (57%) after a median of 40 days from disease onset (IQR 29-51, range 21-96). 75% of patients older than 12 years received second-line treatment, compared to 48% of those younger than 12 years. Rituximab was administered to all 20 patients who received second-line immunotherapy. Cyclophosphamide was used in one patient and was administered prior to rituximab. Four patients received bortezomib, of whom only one received it before rituximab. The median interval between the first second-line treatment and a successive escalating agent was 47 days (IQR 44-48). Full immunotherapy details are provided in Supplementary Table 1.

### 3.4 Symptomatic treatments

#### 3.4.1 Antipsychotics

**Fig. 5:**
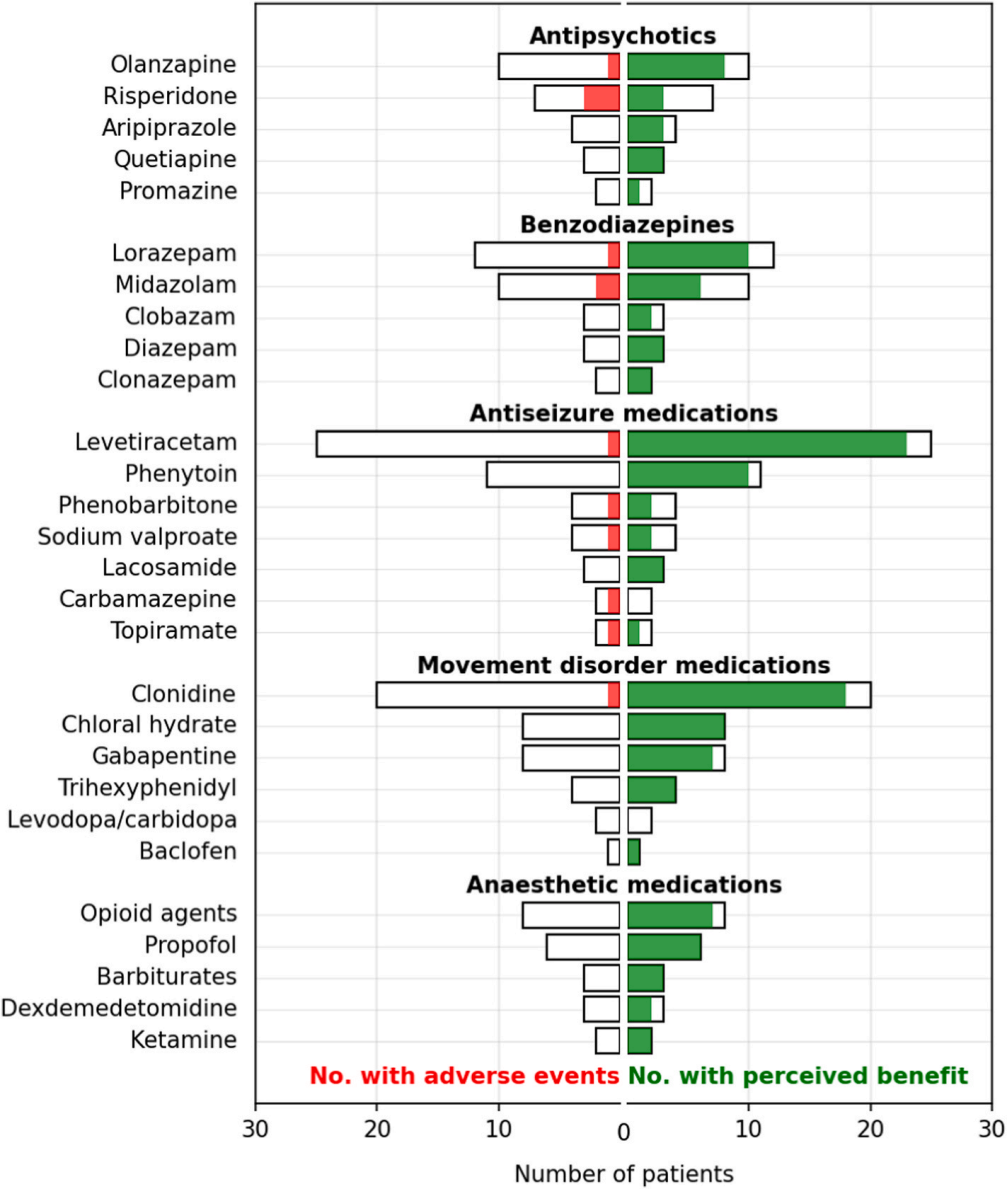
Adverse and beneficial effects of symptomatic medications. Empty bars show the total number of patients who received each medication, filled red for those with adverse effects and green for those with with perceived benefit. (For interpretation of the references to color in this figure legend, the reader is referred to the Web version of this article.)

Antipsychotics were administered to 17 patients (49%) to manage psychiatric symptoms and agitation, with a median treatment duration of 110 days (IQR 92-151) and an adverse event:benefit ratio of 23%:94% (0.24) (Fig. 5). Second-generation antipsychotics were predominantly used, with olanzapine administered in 10 patients (29%), risperidone in 7 (20%), aripiprazole in 4 (11%) and quetiapine in 3 (9%). Promazine, administered to 2 patients (6%), was the only first-generation antipsychotic. Risperidone had the highest adverse event:benefit ratio (43%:43% [1]), with three patients suffering moderate severity (CTCAE grade 2) adverse effects (akathisia, hyperprolactinemia and insomnia), leading to discontinuation in the patient with akathisia. Olanzapine, aripiprazole, and quetiapine were generally well tolerated, with only one olanzapine recipient developing mild drowsiness (CTCAE grade 1) that resolved without intervention.

#### 3.4.2 Benzodiazepines

Benzodiazepines were used in 21 patients (60%), most frequently lorazepam (n = 12; 34%) and midazolam (n = 10, 29%), followed by clobazam and diazepam (each n = 3; 9%) and clonazepam (n = 2; 6%). Benzodiazepines were administered to control different symptoms such as seizures, agitation, movement disorders and sleep disturbances, and for ICU sedation, with a median treatment duration of 49 days (IQR 4-79), and adverse event:benefit ratio 14%:90% (0.16). Adverse events occurred in one lorazepam recipient and two midazolam recipients. One patient receiving midazolam experienced severe respiratory depression (CTCAE grade 3) after intravenous bolus administration in the ICU, leading to discontinuation.

#### 3.4.3 Anti-seizure medications

Anti-seizure medications were the most frequently used category, prescribed to 28 patients (80%) with a median duration of 183 days (IQR 130-306) and an adverse event:benefit ratio of 18%:96% (0.19). Levetiracetam (n = 25; 71%) was the most common, followed by phenytoin (n = 11; 31%), phenobarbitone and sodium valproate (each n = 4, 11%), lacosamide (n = 3; 9%), and carbamazepine and topiramate (each n = 2; 6%). Levetiracetam showed good efficacy with an adverse event:benefit ratio of 0.04 (4%:92%).

#### 3.4.4 Movement disorder drugs

Movement disorder drugs (MDDs) were given to 21 patients (60%), including clonidine (n = 20; 57%), chloral hydrate and gabapentin (each n = 8; 23%), trihexyphenidyl (n = 4; 11%), levodopa/carbidopa (n = 2; 6%), and baclofen (n = 1; 3%). Median time on MDDs was 92 days (IQR 61-242); adverse event:benefit ratio was 5%:95% (0.05). Among these agents chloral hydrate and clonidine were not used exclusively for movement disorder control but also for agitation, restlessness and sedation induction. MDDs demonstrated a high rate of perceived benefit and were generally well tolerated with only one mild adverse event (CTCAE grade 1, drooling) reported for clonidine.

#### 3.4.5 Anaesthetics agents

Anaesthetics were reported to be administered to 11 patients (31%) and included opioid agents (n = 8, 23%), propofol (n = 6; 17%), dexmedetomidine and barbiturates (n = 3 each; 9%) and ketamine (n = 2; 6%). No adverse events were reported in this category, though interpretation is limited by critical illness context in ICU recipients and possibility of underreporting. Full details of all symptomatic treatments are provided in Supplementary Table 2.

### 3.5 Clinical outcomes and symptom persistence

The median length of hospital stay was 60 days (interquartile range [IQR] 30-89). At the time of discharge only 12 patients (34%) had achieved a good functional recovery (mRS 0-1), improving to 21 (68%) at one year and 31 (87%) at last follow-up (median 5 years [range 1-13]). PASS total scores followed a similar trajectory to mRS (Fig. 3), with significant improvements from nadir (median 22) to discharge (median 6 [IQR 4–8, range 0–22]), followed by smaller incremental improvements at one year (median 3 [IQR 1–5, range 0–20]), and last follow-up (median 0 [IQR 0–2, range 0–14]) (Supplementary Table 3). Six patients younger than 12 years (17%) had a relapsing course, compared to none of the older patients (p = 0.07).

PASS item analysis highlighted patterns of long-term symptom persistence (Supplementary Figure) despite overall good functional recovery. One year after disease onset, 12/35 (34%) patients were still on antiseizure medications (seizures controlled in 10/12 [83%]), 11/35 (31%) still had psychiatric symptoms (median duration 126 days [IQR 85-453]) and 8/35 (23%) still had movement disorders (median duration 112 days [IQR 60-361]) (Fig. 4). By last follow-up the frequencies of antiseizure medications, psychiatric symptoms and movement disorders had all improved to 4/35 (11%); the most persistent impairments were in activities of daily living (9/35, 26%), speech/communication (6/35, 17%), and sleep (5/35, 14%).

## 4 Discussion

In this bicentric paediatric NMDAR antibody encephalitis cohort, we provide a symptom-by-symptom characterisation of disease onset, progression, and long-term persistence using the novel PASS score, alongside a pragmatic overview of symptom-directed pharmacological management. Early phenotype differed by age at onset, but disease severity converged at nadir in both adolescent and younger patients. Our findings are consistent with the observation that recovery in NMDARE tends to occur in the reverse order of symptom presentation (Kayser M and Dalmau, 2011), with later-appearing symptoms such as movement disorders and dysautonomia resolving earlier, and earlier-appearing manifestations such as cognitive and psychiatric symptoms tending to persist longer (Liu et al., 2019). Despite favourable global functional outcomes in most patients, persistent neurocognitive and functional impairments affecting daily activities were common at long-term follow-up, particularly involving speech and communication and sleep. In this context, PASS captured clinically meaningful differences across both acute and chronic phases that were not adequately reflected by global disability measures alone. Symptom-directed treatments were generally perceived as effective and well tolerated in routine care, including second-generation antipsychotics, with the notable exception of risperidone, which showed a less favourable profile in this cohort.

### 4.1 Symptom evolution and outcome

Symptom evolution mapping in our paediatric NMDARE cohort revealed several consistencies with previous studies (Titulaer et al., 2013). Seizures and psychiatric manifestations were the most frequent symptoms at disease onset (Irani et al., 2010). Age at onset shaped the early clinical phenotype (Armangue et al., 2013): children <12 years showed broader and more functionally disruptive symptoms, whereas adolescents predominantly presented with psychiatric agitation and fewer neurological signs. Notably, no patients aged 12 years or older presented with fever or neurological symptoms such as movement disorders, motor deficits, or gait instability at onset. These age-related differences in clinical presentation at disease onset are probably responsible for the slightly later hospital admission in the older age group.

Subsequent symptom evolution tended to be similar for both age groups, with high disease severity at nadir, reflected by a median mRS score of 5 and a median of nine symptoms (Shim et al., 2020). Despite this illness burden, almost 90% of patients achieved excellent functional recovery (mRS 0-1). This likely reflects the efficacy of prompt immunotherapy which has been correlated with favourable outcomes (Nosadini et al., 2021b). However, previous studies have highlighted that despite an overall good functional recovery, cognitive deficits may persist years after encephalitis (Flet-Berliac et al., 2023; Cainelli et al., 2019; Matricardi et al., 2016). In our cohort, PASS item analysis showed that more than 25% of patients had persistent impairments in the safety and age-appropriate activities of daily living domain at last follow-up. A recent study reported that up to 68% of the patients had unfavourable recovery at 2 years follow-up, including 54% with cognitive impairment (Mazowiecki et al., 2025). These findings underscore that a substantial proportion of patients do not return to a fully satisfactory quality of life or achieve complete recovery to premorbid functioning. The discrepancy between a good global outcome on the mRS and persistent deficits in daily functioning likely reflects the subtle nature of many residual symptoms, which may not significantly affect mRS scores. As the mRS primarily assesses motor function, it may fail to capture neuropsychological domains that measure participation, cognition, and quality of life (De Bruijn et al., 2018; Brenner et al., 2024). The most frequent persistent symptoms at last follow-up in our cohort were speech and communication dysfunction and sleep disturbances. These domains, which are difficult to capture in mRS scoring, can significantly affect quality of life and expected developmental trajectories in children.

Sleep disturbances such as agitated insomnia (Gurrera, 2019) and sleep-wake reversal (Al-Diwani et al., 2026) are hallmarks of NMDARE in the acute phase and may be helpful in distinguishing the disorder from primary psychiatric conditions or infectious encephalitis (Bean et al., 2025). After the acute phase, which may last several weeks, sleep patterns typically shift, with many patients developing hypersomnia that can persist for months (and occasionally years) despite tapering or discontinuation of sedative medications (Ariño et al., 2020). This transition from insomnia to hypersomnia is consistent with the broader clinical evolution of NMDARE, in which the active inflammatory phase - characterised by predominantly positive symptoms such as psychosis, dyskinesias, and autonomic dysfunction - gives way to a recovery phase marked by negative symptoms and executive dysfunction (Muñoz-Lopetegi et al., 2020). Persistent sleep disturbances may have important implications for neurodevelopment, as sleep plays a critical role in brain maturation and cognitive development across childhood, supporting processes such as memory consolidation, learning, and emotional regulation. Disrupted or insufficient sleep during development has been associated with impairments in cognitive performance, behaviour, and emotional functioning, potentially influencing neurodevelopmental trajectories (Mason et al., 2021).

A previous study (Shim et al., 2020) using the CASE scoring system in paediatric NMDARE highlighted difficulty in differentiating language and memory deficits in younger patients and emphasized the need for revised criteria in paediatric populations. In our cohort, the PASS score detected abnormalities in these domains even in younger children, suggesting its potential utility in identifying subtle yet clinically meaningful impairments that other scales may overlook. The ability of PASS to provide finer resolution across the disease course represents an important advantage in this context. In contrast, mRS showed limited discriminative ability, with many patients clustering at the highest scores during the acute phase and at the lowest scores during recovery, reflecting ceiling and floor effects (Fig. 3). By capturing a broader range of symptom domains and severities, PASS maintained greater sensitivity to clinical variation both at peak disease and during recovery, allowing a more detailed characterisation of disease burden and recovery trajectories in paediatric NMDARE.

Interestingly, all patients in our cohort who experienced a relapse were younger than 12 years. This observation appears to differ from previous findings, where adolescent age was associated with an increased risk of relapsing disease in multivariable analyses controlling for treatment exposure (Nosadini et al., 2021b). In our cohort, however, older children more frequently received second-line immunotherapy, which has been associated with a reduced risk of relapse (Nosadini et al., 2021b; Seery et al., 2025). The broader use of second-line immunotherapy in adolescents may partly explain the lower relapse frequency observed in this age group.

### 4.2 Symptomatic treatments

While immunotherapy is the cornerstone of treatment in NMDARE and key determinant of both symptom resolution and long-term outcome (Titulaer et al., 2013; Nosadini et al., 2021a, 2021b; Eyre et al., 2020), the often prolonged interval between immunotherapy initiation and clinical response means that symptom-directed pharmacological management plays an essential adjunctive role during the acute and subacute phases. Effective symptomatic control can reduce morbidity and distress, support safety and sleep, and facilitate access to rehabilitation therapies (Mohammad et al., 2016).

In adults, the use of antipsychotic medications in NMDARE has been controversial, with studies reporting high susceptibility to adverse events and severe complications, including neuroleptic malignant syndrome (NMS) or worsening of movement disorder (Lejuste et al., 2016); however, differentiating between NMS and disease-related symptoms can be challenging (Dalmau et al., 2019). Autonomic instability, decreased level of consciousness, and dystonic posturing with increased blood concentration of creatine kinase or rhabdomyolysis can occur during the natural course of the encephalitis, even in patients not treated with antipsychotics, particularly in the ICU setting (Schwarz et al., 2023). Other reports have not confirmed this increased susceptibility, describing beneficial effects of antipsychotics, especially for agitation control (Warren et al., 2021). A previous study in children also reported increased vulnerability to antipsychotic-related adverse effects in NMDARE (Mohammad et al., 2016), however the patients in this study mainly received first-generation antipsychotics and high-dose dopamine blockade agents such as haloperidol and risperidone. In our cohort, no cases of NMS occurred. Adverse events were mostly associated with risperidone use, with three moderate (CTCAE grade 2) adverse events out of seven patients, only one of which was movement disorder–related (akathisia). NMDAR hypofunction can disrupt the regulation of midbrain dopaminergic neurons through effects on inhibitory circuits, contributing to abnormal dopaminergic transmission and increased striatal dopamine activity (Dwyer et al., 2024). Since antipsychotic adverse effects are closely related to dopamine D2 receptor blockade and increase when receptor occupancy exceeds the therapeutic window (Siafis et al., 2023), such dopaminergic dysregulation may increase vulnerability to strong D2 antagonism. Consistent with this framework, the other antipsychotics administered in our cohort (olanzapine, aripiprazole, and quetiapine) were generally well tolerated and associated with a favourable perceived benefit, possibly reflecting their lower effective dopamine receptor blockade (Kayser M and Dalmau, 2011; Abboud et al., 2021).

Benzodiazepines have been used for management of a broad range of symptoms in NMDARE, including agitation, sleep disorders, seizures, movement disorders and catatonia (Mohammad et al., 2016; Eyre et al., 2020; Kruse et al., 2014). In our cohort benzodiazepines had a generally good adverse event:benefit ratio, with just one discontinuation due to adverse effects (respiratory depression in an ICU patient). Although generally safe and effective, critically ill patients receiving benzodiazepines require close monitoring (Neyens et al., 2018).

Movement disorder drugs were also widely used in our cohort, with good tolerability. Clonidine was the most frequent, administered both for movement disorder control and for sedation and sleep regulation (Mohammad et al., 2016). Anti-seizure medications were the most frequently prescribed symptomatic drug category, reflecting the high seizure burden in NMDARE patients (Yeshokumar et al., 2021). Multiple agents were used in our cohort, illustrating the heterogeneity of seizure management approaches in this condition (Du et al., 2023). Levetiracetam was the most frequently used. Due to the limited number of patients receiving carbamazepine, our data could not confirm its previously reported superiority over other agents (de Bruijn et al., 2019).

We also collected data on anaesthetic medication use, which was reported as safe in all patients. However, these data should be interpreted cautiously due to the critical condition of these patients, the presence of overlapping symptoms, and the possibility of underreporting adverse effects. Nevertheless, our findings are consistent with the existing literature on anaesthetic use in ICU settings for NMDARE patients (De Montmollin et al., 2017; Schwarz et al., 2023; Neyens et al., 2018).

### 4.3 Limitations

This study has several limitations. The retrospective design and relatively small sample size from two tertiary referral centres may limit generalisability and introduce selection bias. The limited number of patients, and of poor-outcome events in particular, precluded multivariable adjustment; analyses of the relationships between timing of immunotherapy and outcome were therefore univariate and underpowered, and cannot exclude a clinically meaningful association or account for the tendency of more severely affected children to be treated sooner. Evaluation of symptomatic treatment effects relied on retrospective review of clinician documentation rather than predefined prospective outcome measures; therefore, the perceived benefit of medications should be interpreted cautiously. Persisting symptoms were scored using PASS based on family report and clinician documentation rather than formal neuropsychological evaluations, hence long-term cognitive and psychiatric symptoms may be underestimated. Adverse effects of medications may also have been under-reported, particularly in critically ill patients receiving multiple medications in the ICU, where attribution of side effects to individual drugs is challenging.

## 5 Conclusion

In our paediatric NMDARE cohort, early clinical phenotype varied by age at onset but converged to similar severity at nadir. Despite favourable global functional recovery in most patients, persistent neurocognitive and functional deficits affecting daily activities remained common, with speech and communication difficulties and sleep disturbances the most frequent. These residual deficits were poorly captured by mRS, whereas PASS scoring enabled characterisation of subtle but clinically meaningful differences in both acute disease severity and long-term symptoms. Symptom-directed pharmacological treatments demonstrated good overall efficacy and favourable tolerability in routine care. In particular, second-generation antipsychotics with lower dopamine receptor blockade appeared to have a better safety profile for the management of agitation and psychiatric symptoms, whereas risperidone showed a less favourable tolerability pattern in this cohort. Future studies should prospectively and longitudinally apply multidomain severity scores such as PASS throughout the acute and recovery phases of NMDARE, which could enable more precise characterisations of symptom trajectories, rehabilitation needs and treatment effects.

## CRediT authorship contribution statement

**Alessandro Santagostino Barbone:** Conceptualization, Data curation, Formal analysis, Investigation, Methodology, Project administration, Validation, Visualization, Writing – original draft, Writing – review & editing. **Yoshua Collins-Sawaragi:** Data curation, Formal analysis, Investigation, Methodology, Writing – review & editing. **Maria Margherita Mancardi:** Conceptualization, Data curation, Formal analysis, Investigation, Methodology, Supervision, Validation, Visualization, Writing – original draft, Writing – review & editing. **Roberta Biddeci:** Data curation, Investigation, Methodology, Validation, Visualization, Writing – review & editing. **Chiara Scaroni:** Data curation, Investigation, Methodology, Validation, Visualization, Writing – review & editing. **Claire Thompson:** Data curation, Investigation, Methodology, Validation, Visualization, Writing – review & editing. **Silvia Buratti:** Conceptualization, Investigation, Methodology, Supervision, Validation, Writing – original draft, Writing – review & editing. **Lino Nobili:** Conceptualization, Formal analysis, Investigation, Methodology, Validation, Writing – original draft, Writing – review & editing. **Ming Lim:** Conceptualization, Data curation, Formal analysis, Investigation, Methodology, Supervision, Validation, Visualization, Writing – original draft, Writing – review & editing. **Elisa De Grandis:** Conceptualization, Data curation, Formal analysis, Investigation, Methodology, Supervision, Validation, Visualization, Writing – original draft, Writing – review & editing. **Thomas Rossor:** Conceptualization, Data curation, Formal analysis, Investigation, Methodology, Supervision, Validation, Visualization, Writing – original draft, Writing – review & editing. **Michael Eyre:** Conceptualization, Data curation, Formal analysis, Investigation, Methodology, Supervision, Validation, Visualization, Writing – original draft, Writing – review & editing.

## Declaration of competing interest

The authors declare that they have no known competing financial interests or personal relationships that could have appeared to influence the work reported in this paper.

## Figures and Tables

**figs1:**
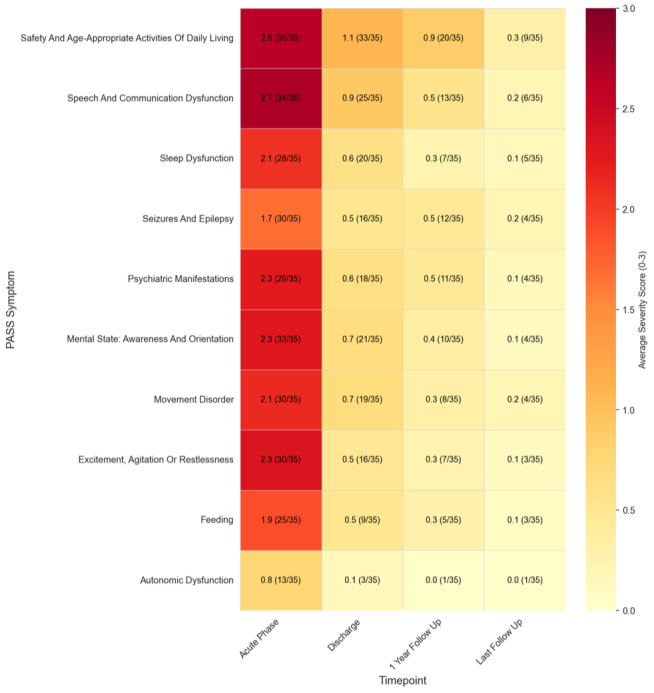


## Data Availability

Deidentified participant data not included in the article are available on request by any qualified investigator to the corresponding author.
