# Supplementary material for: Symptom evolution, persistence, and pharmacological management in paediatric NMDA receptor antibody encephalitis
Source: Brain Behav Immun Health. 2026 Jul 31;56:101320. doi: 10.1016/j.bbih.2026.101320 (PMC13475692; doi:10.1016/j.bbih.2026.101320)
Supplement: Supplementary file 1 [file mmc1.docx]

**Supplementary Table 1:** *Immune treatment.*

| **Characteristic** | **Patients <12 years (n=23)** | **Patients ≥ 12 years (n=12)** | **All Patients (n=35)** |
| --- | --- | --- | --- |
| **Acute phase immunotherapy** | 22 (96%) | 11 (92%) | 33 (94%) |
| **First treatment administration (days from onset)** |  |  |  |
| Mean (SD) | 19 (17) | 22 (10) | 20 (15) |
| Median (IQR) | 14 (9-19) | 19 (16-27) | 16 (10-25) |
| Range | 6 - 83 | 8 - 40 | 6 - 83 |
| **IV steroids** | 18 (78%) | 9 (75%) | 27 (77%) |
| **IV steroids duration (days)** |  |  |  |
| Mean (SD) | 5 (1) | 5 (1) | 5 (1) |
| Median (IQR) | 5 (3-5) | 5 (5-5) | 5 (3-5) |
| Range | 3 - 7 | 3 - 7 | 3 - 7 |
| **Oral steroids acute phase (no tapering)** | 4 (17%) | 0 (0%) | 4 (11%) |
| **Oral steroids duration (days)** |  |  |  |
| Mean (SD) | 4 (1) | N/A | 4 (1) |
| Median (IQR) | 4 (3-4) | N/A | 4 (3-4) |
| Range | 3 - 5 | N/A | 3 - 5 |
| **IVIg** | 18 (78%) | 11 (92%) | 29 (83%) |
| **IVIg duration (days)** |  |  |  |
| Mean (SD) | 3 (1) | 2 (1) | 3 (1) |
| Median (IQR) | 2 (2-5) | 2 (2-2) | 2 (2-4) |
| Range | 2 - 5 | 1 - 5 | 1 - 5 |
| **PLEX** | 11 (48%) | 6 (50%) | 17 (49%) |
| **PLEX duration (days/courses)** |  |  |  |
| Mean (SD) | 5 (2) | 5 (0) | 5 (1) |
| Median (IQR) | 5 (4-5) | 5 (5-5) | 5 (5-5) |
| Range | 2 - 7 | 5 - 5 | 2 - 7 |
| **Oral steroid tapering 3-6 months** | 8 (35%) | 1 (8%) | 9 (26%) |
| **Oral steroid tapering 6-12 months** | 2 (9%) | 0 (0%) | 2 (6%) |
| **Oral steroid tapering < 3 months** | 8 (35%) | 8 (67%) | 16 (46%) |
| **Maintenance immunotherapy (excluding steroids tapering)** | 8 (35%) | 3 (25%) | 11 (31%) |
| **Maintenance immunotherapy everolimus** | 2 (9%) | 1 (8%) | 3 (9%) |
| **Maintenance immunotherapy IVIG cycles** | 1 (4%) | 2 (17%) | 3 (9%) |
| **Maintenance immunotherapy mycophenolate** | 3 (13%) | 0 (0%) | 3 (9%) |
| **Maintenance immunotherapy steroids cycles** | 2 (9%) | 0 (0%) | 2 (6%) |
| **Second line treatment** | 11 (48%) | 9 (75%) | 20 (57%) |
| **Second line treatment rituximab** | 11 (48%) | 9 (75%) | 20 (57%) |
| **Second line treatment cyclophosphamide** | 0 (0%) | 1 (8%) | 1 (3%) |
| **Second line treatment bortezomib** | 3 (13%) | 1 (8%) | 4 (11%) |
| **Time to second line treatment (days from onset)** |  |  |  |
| Mean (SD) | 43 (23) | 45 (15) | 44 (19) |
| Median (IQR) | 39 (26-54) | 42 (35-48) | 40 (29-51) |
| Range | 21 - 96 | 29 - 75 | 21 - 96 |

*Abbreviations: SD standard deviation; IQR interquartile range; IV intravenous; IVIG intravenous immunoglobulins; PLEX: plasma exchange. Data are n (n %) or mean (SD) median (IQR), range*

**Supplementary Table 2:** *symptomatic treatment*

| **Medication** | **Patients n(%)** | **Duration (days) Median (IQR)** | **Duration (days) Range** | **Perceived Benefit n(%)** | **Adverse Event n(%)** | **Adverse Event:Benefit Ratio** | **Adverse Event detail (CTCAE grade)** |
| --- | --- | --- | --- | --- | --- | --- | --- |
| **Antipsychotics** | 17 (49%) | 110 (20-151) | 11 - 365 | 16 (94%) | 4 (24%) | 4:16 (0.25) |  |
| Promazine | 2 (6%) | 44 (33-54) | 23 - 64 | 1 (50%) | 0 (0%) | 0:1 (0.00) |  |
| Aripiprazole | 4 (11%) | 58 (6-136) | 1 - 212 | 3 (75%) | 0 (0%) | 0:3 (0.00) |  |
| Olanzapine | 10 (29%) | 94 (19-117) | 14 - 365 | 8 (80%) | 1 (10%) | 1:8 (0.12) | Drowsiness (Grade 1) |
| Quetiapine | 3 (9%) | 101 (72-112) | 42 - 122 | 3 (100%) | 0 (0%) | 0:3 (0.00) |  |
| Risperidone | 7 (20%) | 35 (9-167) | 3 - 335 | 3 (43%) | 3 (43%) | 3:3 (1.00) | Akathisia (Grade 2), Hyperprolactinemia (Grade 2), Insomnia (Grade 2) |
| **Benzodiazepines** | 21 (60%) | 49 (8-140) | 1 - 225 | 19 (90%) | 3 (14%) | 3:19 (0.16) |  |
| Clobazam | 3 (9%) | 121 (90-152) | 60 - 182 | 2 (67%) | 0 (0%) | 0:2 (0.00) |  |
| Clonazepam | 2 (6%) | 39 (39-39) | 39 - 39 | 2 (100%) | 0 (0%) | 0:2 (0.00) |  |
| Diazepam | 3 (9%) | 14 (8-120) | 1 - 225 | 3 (100%) | 0 (0%) | 0:3 (0.00) |  |
| Lorazepam | 12 (34%) | 50 (2-90) | 1 - 151 | 10 (83%) | 1 (8%) | 1:10 (0.10) | Drowsiness (Grade 2) |
| Midazolam | 2 (6%) | 4 (2-6) | 0 - 38 | 6 (50%) | 2 (20%) | 2:6 (0.33) | Sedation (Grade 2), Respiratory Depression (Grade 3) |
| **Anti-Seizure Medications** | 28 (80%) | 184 (130-306) | 3 - 918 | 27 (96%) | 5 (18%) | 5:27 (0.18) |  |
| Carbamazepine | 2 (6%) | 5 (4-6) | 3 - 7 | 0 (0%) | 1 (50%) | N/A | Leukopenia (Grade 2) |
| Lacosamide | 3 (9%) | 456 (349-563) | 242 - 670 | 3 (100%) | 0 (0%) | 0:3 (0.00) |  |
| Levetiracetam | 25 (71%) | 184 (152-374) | 23 - 918 | 23 (92%) | 1 (4%) | 1:23 (0.04) | Irritability (Grade 2) |
| Phenobarbital | 4 (11%) | 19 (3-89) | 7 - 273 | 2 (50%) | 1 (25%) | 1:2 (0.50) | Sedation (Grade 2) |
| Phenytoin | 4 (11%) | 1 (1-2) | 1 - 12 | 10 (91%) | 0 (0%) | 0:10 (0.00) |  |
| Sodium Valproate | 4 (11%) | 72 (38-127) | 4 - 182 | 2 (50%) | 1 (25%) | 1:2 (0.50) | Increase of liver enzymes (Grade 1) |
| Topiramate | 2 (6%) | 14 (12-15) | 10 - 17 | 1 (50%) | 1 (50%) | 1:1 (1.00) | Paraesthesia (Grade 1) |
| **Movement Disorder Drugs** | 21 (60%) | 92 (61-242) | 13 - 853 | 20 (95%) | 1 (5%) | 1:20 (0.05) |  |
| Baclofen | 1 (3%) | 30 (30-30) | 30 - 30 | 1 (100%) | 0 (0%) | 0:1 (0.00) |  |
| Chloral hydrate | 8 (23%) | 65 (56-122) | 9 - 191 | 8 (100%) | 0 (0%) | 0:8 (0.00) |  |
| Clonidine | 20 (57%) | 86 (28-165) | 13 - 792 | 18 (90%) | 1 (5%) | 1:18 (0.06) | Drooling (Grade 1) |
| Gabapentin | 8 (23%) | 118 (61-378) | 18 - 853 | 7 (88%) | 0 (0%) | 0:7 (0.00) |  |
| Levodopa/  Carbidopa | 2 (6%) | 16 (10-21) | 5 - 26 | 0 (0%) | 0 (0%) | N/A |  |
| Trihexyphenidyl | 4 (11%) | 255 (152-320) | 25 - 365 | 4 (100%) | 0 (0%) | 0:4 (0.00) |  |
| **Anaesthetics** | 11 (31%) | 17 (10-72) | 1 - 115 | 10 (91%) | 0 (0%) | 0:10 (0.00) |  |
| Barbiturates | 3 (9%) | 52 (37-84) | 22 - 115 | 3 (100%) | 0 (0%) | 0:3 (0.00) |  |
| Dexmedetomidine | 3 (9%) | 8 (8-12) | 8 - 17 | 2 (67%) | 0 (0%) | 0:2 (0.00) |  |
| Ketamine | 2 (6%) | 14 (13-16) | 12 - 17 | 2 (100%) | 0 (0%) | 0:2 (0.00) |  |
| Opioid agents | 8 (23%) | 19 (11-66) | 4 - 98 | 7 (88%) | 0 (0%) | 0:7 (0.00) |  |
| Propofol | 6 (17%) | 7 (2-12) | 1 - 21 | 6 (100%) | 0 (0%) | 0:6 (0.00) |  |

*Abbreviations: CTCAE Common Terminology Criteria for Adverse Events v 5.0. Data are n (n %) or median (IQR), range.*

**Supplementary Table 3:** *Clinical outcome.*

| **Patient Characteristics** | **All Patients (n=35)** | **Patients <12 years (n=23)** | **Patients ≥ 12 years (n=12)** |
| --- | --- | --- | --- |
| **mRS nadir** |  |  |  |
| Mild Severity (mRS 0-1) | 0 (0%) | 0 (0%) | 0 (0%) |
| Moderate-High Severity (mRS 2-6) | 35 (100%) | 23 (100%) | 12 (100%) |
| Mean (SD) | 4 (1) | 4 (1) | 4 (1) |
| Median (IQR) | 5 (4-5) | 4 (4-5) | 5 (4-5) |
| Range | 2 - 5 | 2 - 5 | 2 - 5 |
| **PASS scale total score (0 - 30) nadir** |  |  |  |
| Mean (SD) | 21 (7) | 20 (7) | 22 (7) |
| Median (IQR) | 22 (16-26) | 21 (15-26) | 24 (18-26) |
| Range | 5 - 30 | 5 - 30 | 8 - 30 |
| **mRS discharge** |  |  |  |
| Good Outcome (mRS 0-1) | 12 (34%) | 7 (30%) | 5 (42%) |
| Bad Outcome (mRS 2-6) | 23 (66%) | 16 (70%) | 7 (58%) |
| Mean (SD) | 2 (1) | 2 (1) | 2 (1) |
| Median (IQR) | 2 (1-2) | 2 (1-2) | 2 (1-2) |
| Range | 0 - 4 | 0 - 3 | 1 - 4 |
| **PASS scale total score (0 - 30) discharge** |  |  |  |
| Mean (SD) | 6 (4) | 6 (3) | 6 (5) |
| Median (IQR) | 6 (4-8) | 7 (4-8) | 6 (5-6) |
| Range | 0 - 22 | 0 - 12 | 2 - 22 |
| **mRS 1 year** |  |  |  |
| Good Outcome (mRS 0-1) | 21 (68%) | 13 (62%) | 8 (80%) |
| Bad Outcome (mRS 2-6) | 10 (32%) | 8 (38%) | 2 (20%) |
| Mean (SD) | 1 (1) | 1 (1) | 1 (1) |
| Median (IQR) | 1 (1-2) | 1 (1-2) | 1 (0-1) |
| Range | 0 - 4 | 0 - 3 | 0 - 4 |
| **PASS scale total score (0 - 30) 1 year** |  |  |  |
| Mean (SD) | 4 (4) | 4 (3) | 3 (6) |
| Median (IQR) | 3 (1-6) | 4 (2-6) | 2 (0-3) |
| Range | 0 - 20 | 0 - 10 | 0 - 20 |
| **mRS at last follow up** |  |  |  |
| Good Outcome (mRS 0-1) | 31 (89%) | 21 (91%) | 10 (83%) |
| Bad Outcome (mRS 2-6) | 4 (11%) | 2 (9%) | 2 (17%) |
| Mean (SD) | 1 (1) | 0 (1) | 1 (1) |
| Median (IQR) | 0 (0-1) | 0 (0-1) | 0 (0-1) |
| Range | 0 - 4 | 0 - 2 | 0 - 4 |
| **PASS scale total score (0 - 30) last follow up** |  |  |  |
| Mean (SD) | 2 (3) | 1 (2) | 2 (4) |
| Median (IQR) | 0 (0-2) | 0 (0-2) | 0 (0-2) |
| Range | 0 - 14 | 0 - 7 | 0 - 14 |

*Abbreviations: mRS, modified Rankin Scale; PASS, Paediatric Autoimmune Encephalitis Severity Score. Data are n (n %) or mean (SD) median (IQR), range*

**Supplementary Table 4:** *Post hoc analysis – time to first immunotherapy and clinical outcome (n = 33).*

|  | **Good outcome (mRS 0–1) n = 29** | **Poor outcome (mRS 2–6) n = 4** | **p-value** |
| --- | --- | --- | --- |
| **Analysis 1 – Mann–Whitney U: days to first immunotherapy vs mRS outcome at last follow-up** | | | |
| **Days to first immunotherapy** |  |  |  |
| Median (IQR) | 16 (10–25) | 17 (12.8–25.5) |  |
| Range | 6–83 | 15–40 |  |
| Mann–Whitney U statistic | 58 |  |  |
| p-value | 1.000 |  | ns |
| **Analysis 2 – Mann–Whitney U: early (≤14 days) vs late (>14 days) immunotherapy** | | | |
|  | **Early IT (≤14 days) n = 13** | **Late IT (>14 days) n = 20** | **p-value** |
| **2a. PASS score at last follow-up** |  |  |  |
| Median (IQR) | 0 (0–4) | 0 (0–1.2) |  |
| Mann–Whitney U statistic | 140 |  |  |
| p-value | 0.676 |  | ns |
| **2b. Hospital stay duration (days)** |  |  |  |
| Median (IQR) | 71 (30–118) | 55.5 (42.5–69) |  |
| Mann–Whitney U statistic | 144 |  |  |
| p-value | 0.632 |  | ns |
| **Analysis 3 – Spearman rank correlation: days to first immunotherapy** | | | |
| **Correlation** | **n** | **Spearman rₛ** | **p-value** |
| 3a. Days to IT vs PASS at last follow-up | 33 | -0.157 | 0.382 (ns) |
| 3b. Days to IT vs hospital stay duration | 33 | -0.217 | 0.224 (ns) |

*Abbreviations: IT, immunotherapy; IQR, interquartile range; mRS, modified Rankin Scale; PASS, Paediatric Autoimmune Encephalitis Severity Score; ns, not significant (p ≥ 0.05). Analysis 2 uses a 14-day clinically meaningful cut-off. Analyses 3a and 3b used Spearman rank correlation (two-tailed).*

**Supplementary Figure**

*PASS score heatmap at different timepoints. Each row shows a different subitem of the scale. Data are represented as n = average (mean) severity score (0–3) and ordered from the most to least persistent at last follow-up (top to bottom). Numbers in brackets indicate the number of patients presenting the symptom out of the total cohort (n = 35).*
